# Bio-Inspired Double-Layered Hydrogel Robot with Fast Response via Thermo-Responsive Effect

**DOI:** 10.3390/ma17153679

**Published:** 2024-07-25

**Authors:** Yunsong Liu, Xiong Zheng

**Affiliations:** 1 College of Mechanical and Vehicle Engineering, Hunan University, Changsha 410082, China; liuyunsongdut@163.com; 2 School of Mechanical Engineering, Dalian University of Technology, Dalian 116081, China

**Keywords:** hydrogel robot, thermo-responsiveness, anisotropic structure, caterpillar

## Abstract

Bio-inspired hydrogel robots have become promising due to their advantage of the interaction safety and comfort between robots and humans, while current hydrogel robots mainly focus on underwater movement due to the hydration–dehydration process of thermo-responsive hydrogels, which greatly limits their practical applications. To expand the motion of the thermo-responsive hydrogel robot to the ground, we constructed a hydrogel robot inspired by a caterpillar, which has an anisotropic double-layered structure by the interfacial diffusion polymerization method. Adding PVA and SA to PNIPAm will cause different conformation transitions. Therefore, sticking the two layers of hydrogel together will form a double-layer anisotropic structure. The ultra-high hydrophilicity of PVA and SA significantly reduces the contact angle of the hydrogel from 53.1° to about 10° and reduces its hydration time. The responsive time for bending 30° of the hydrogel robot has been greatly reduced from 1 h to half an hour through the enhancement of photo-thermal conversion and thermal conductivity via the addition of Fe_3_O_4_ nanoparticles. As a result, the fabricated hydrogel robot can achieve a high moving speed of 54.5 mm·h^−1^ on the ground. Additionally, the fabricated hydrogel has excellent mechanical strength and can endure significant flexibility tests. This work may pave the road for the development of soft robots and expand their applications in industry.

## 1. Introduction

Currently, most robots used in our daily lives are made of rigid materials, and these robots perform various tasks through programmable actions, making important contributions to human technological progress [1,2]. However, traditional rigid robots are limited in their adaptability due to the inclusion of rigid links and joints in their construction [3,4]. There is a significant difference in elastic modulus between the components of robots and biological tissues, which poses certain safety risks when interacting with humans [5,6]. In order to overcome this problem, people began to develop soft robots, which are manufactured using continuously deformable materials with high degrees of freedom, allowing them to handle complex tasks more flexibly than traditional robots [7,8]. At the same time, due to the elastic modulus of these materials, similar to biological tissues, soft robots can safely and comfortably interact with humans, which is of great significance in reducing tissue damage that may occur during interactions with biological tissues or organs [9,10]. Therefore, the development of soft materials is rapidly advancing, opening up new possibilities for the development of robot technology [11,12]. Adopting these flexible materials is expected to further improve the interaction safety and comfort between robots and humans, thereby promoting the development of human–machine cooperation [13].

Thermo-responsive hydrogel has attracted extensive attention in the field of soft robots due to their similarity to biological tissues. This material has sensitive responses to various stimuli and can produce reversible morphological changes [14], and this change is essentially the process of converting internal chemical energy into external mechanical energy [15]. Because hydrogel materials have self-deforming and soft tissue-like mechanical properties, they can imitate biological behavior and are widely used in many fields, including bionic devices and soft robots [16]. In the literature, a variety of simple and powerful soft actuators/robots have been reported, and these designs are based on hydrogel materials.

Wu et al. fabricated an isotropic hydrogel robot that can achieve multi-degree of freedom motion underwater [17]. Wei et al. synthesized a hydrogel robot with a single layer by 3D printing, which has programmable responsiveness by near-infrared irradiance under water [18]. Shiblee et al. fabricated a soft gripper using 3D printing technology, which can successfully grip, transport, and release glass vials in water [19]. In addition, there are also hydrogel robots designed with adaptive and multimodal motion capabilities [20]. Although the existing hydrogel actuators can generate a variety of bionic forms, there are still some scientific challenges before their practical application. First, the existing self-deformable soft robots mainly work under water due to the swelling–collapse transition of thermo-responsive hydrogel. Second, the response time of hydrogel is usually long, which leads to the slow-motion speed of the hydrogel robot. Therefore, comprehensive consideration of material and structure designs is required to solve these issues. 

In nature, organisms have evolved various types of movement, such as walking, crawling, and swimming, to adapt to changes in the external environment, which inspires the design of hydrogel soft robots that can achieve motion through self-deformation and interaction with the external environment. In this paper, inspired by a caterpillar, we developed a double-layered hydrogel robot by the interfacial diffusion polymerization (IDP) method, which can walk on the ground. The fabricated robot has an anisotropic structure and can move quickly through hydrogel bending under light irradiance. The fabricated hydrogel has excellent mechanical strength, good photo-thermal conversion ability, and fast responsive time, and it can achieve a high moving speed of 54.5 mm·h^−1^. The structure of the article is shown below. Section 2 introduces the experimental methods, and Section 3 presents and analyzes the experimental results. Then, Section 4 summarizes the whole work. The main novelty of this work is that we creatively fabricated a hydrogel robot crawling on the ground with high moving speed, which may promote the development of hydrogel robots in academic and industrial areas.

## 2. Experimental Section

### 2.1. Materials

The detailed information of the samples used in this work is presented in Table 1. N-Isopropyl acrylamide (NIPAm, purity is 98%, containing stabilizer MEHQ), polyvinyl alcohol (PVA, purity is 99%), sodium alginate (SA, Analytical Reagent), potassium persulfate (PP, purity is 99%), tetramethylethylenediamine (TEMED, purity is 99%), N,N′-methylenebisacrylamide (BIS, purity is 99%) were supplied by Shanghai Aladdin Biochemical Technology Co., Ltd. (Shanghai, China). Ferroferric oxide (Fe_3_O_4_) nanoparticles with a diameter of 50 nm were purchased from XFNANO Co., Ltd. (Nanjing, China). 

### 2.2. Experimental Design

The driving force for the movement of hydrogel robots is based on the different response characteristics of two hydrogel layers. Therefore, the photothermal response characteristics of two hydrogel layers were studied first. Secondly, the bending characteristics of the two layers of hydrogels bonded together were studied. Third, the response of hydrogels depends on the photo-thermal conversion ability, so the influence of absorbance material concentration and light intensity on response speed was investigated. Finally, after obtaining the optimal conditions, we prepared the hydrogel robot and explored its true moving speed under light conditions. 

### 2.3. Preparation of Hydrogels

Here, three hydrogel samples, namely top layer, bottom layer, and double-layered hydrogel, have been fabricated, and the detailed processes are described as follows. 

First, the top layer is fabricated. First, weigh 2.0 g NIPAm, 15.0 g DI water, and 0.09 g BIS; mix the weighted samples together; stir for 30 min; and put the prepared solution in the ice water bath. Second, weigh and add 0.1 g of PP, 0.2 g of PVA, and 0.1 g of TEMED to the prepared solution conditions and stir evenly. Third, pour the solution into the mold; the solution will slowly polymerize at room temperature, and the mold has dimensions 10 mm in width, 50 mm in length, and 5 mm in depth. After about 10 min, the top layer can be successfully fabricated. Then, we fabricate the bottom layer. The fabrication of the bottom layer is almost the same as that of the top one except for one component that is different: the fabrication of the bottom layer uses 0.2 g SA instead of PVA. 

Meanwhile, IDP technology was used to synthesize the double-layered hydrogel. In IDP technology, first, a hydrogel layer is fabricated; second, the initiator solution is coated on the surface of the prepared hydrogel layer so the initiator diffuses to the precursor of the hydrogel. Gradually, a new layer can grow and firmly attach to the surface of the prepared hydrogel [21]. Polymerization by IDP technology can synthesize hydrogel with better thermal stability and thermo-responsiveness compared with traditional methods like physical crosslinking and carboxymethyl cellulose nano-bonding. The key advantage of IDP technology is that it can realize the customization and functionalization of material structure so that the hydrogel actuator can adapt to different application requirements. In addition, the controllability of the IDP process enables researchers to accurately adjust the thickness, composition, and performance of the new growth layer, providing powerful tools for the development of new intelligent materials and soft robots.

The IDP process for the fabrication of double-layered hydrogel with anisotropic structure is described as follows. First, the bottom layer with the designed shape was synthesized through the above procedures. Second, add 50 mL water and 5 g PP into the bottom layer and let the mixture stand for 10 min. Third, mix 2 g NIPAm, 15 mL water, and 0.9 g BIS together and put the mixture into the ice bath. Fourth, add 0.3 mL TEMED, 0.1 g PP, and 0.2 g PVA into the mixture and pour the as-prepared solution into the mold. Let the mixture stand for 5 min, and the double-layered hydrogel with PNIPAm-SA as bottom layer and PNIPAm-PVA as top layer can be successfully fabricated. These are the fabrication processes of the double-layered hydrogel using IDP method. The prepared double-layered hydrogel has a dimension of 10 mm width, 10 mm thickness, and 50 mm length, respectively, and each layer has a thickness of 5 mm.

### 2.4. Characterization

A universal testing machine (Hongjin, Dongguan, China, LL-05) is applied to measure the stress–strain curve of the hydrogel. A thermometer (Xiangyi, Xiangtan, China, DRL-III) is applied to evaluate the thermal conductivity of the evaporator. The absorptance of the samples is measured by a UV–vis–NIR spectrophotometer (Hitachi, Tokyo, Japan, U4100). The contact angles were detected via a contact angle meter (KRUSS, Hamburg, Germany, DSA100).

## 3. Results and Discussions

### 3.1. Mechanical Strength

**Figure 1:**
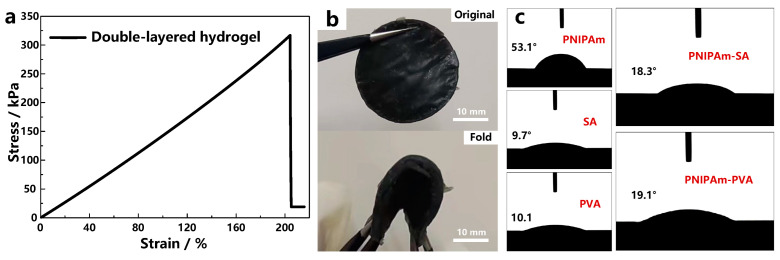
Mechanical performance evaluation of the double-layered hydrogel. (**a**) Stress–strain curve; (**b**) flexibility test. (**c**) Contact angles of different hydrogels.

Mechanical performance is crucial for robots, so the mechanical strength has been evaluated by stress–strain measurement and flexibility tests, shown in Figure 1a,b. The tensile rate is kept at 50 mm/min. The fabricated hydrogel has excellent flexibility, and the fracture strain is as high as 212.5%. Because of its good flexibility, the fabricated hydrogel is able to withstand significant deformation, like stretching, folding, and twisting. Figure 1b indicates that it can withstand a bend of 180° with a bend speed of 60°/s. Meanwhile, it also has a fracture stress of 312 kPa, which ensures the endurance of the hydrogel.

### 3.2. Hydrophilicity of Hydrogels

The recovery of the hydrogel robot highly relies on its water absorption capacity, so the contact angles of the hydrogel components to water are systematically measured (Figure 1c). Pure PNIPAm hydrogel has a relatively high contact angle of 53.1°, which indicates that the hydrophilicity of PNIPAm hydrogel is not good. While the contact angles of SA and PVA hydrogels are as low as 9.7° and 10.1°, it is expected that the addition of SA or PVA hydrogels can significantly improve the hydrophilicity of PNIPAm hydrogel [22]. Figure 1c shows that the contact angles of PNIPAm hydrogels are reduced to 18.3° and 19.1° with the addition of SA and PVA hydrogels, respectively.

### 3.3. Influence of Crosslinking Degree

We adjusted the crosslinking degree of hydrogels by changing the concentration of BIS (crosslinker) during the synthesis of hydrogels. The crosslinking degree may influence two aspects of the hydrogels: one is the mechanical strength, and the other is the response time. Three BIS concentrations of 2.6, 3.6, and 4.6 wt% were chosen in the synthesis of hydrogels. When BIS concentrations vary from 2.6 to 4.6 wt%, the fracture stresses of the hydrogels are 183, 312, and 321 kPa, respectively. A too-low crosslinking degree results in a too-weak mechanical strength, and the trend of strength increasing with the crosslinking degree becomes very small when it reaches a certain level. Meanwhile, with the increase in BIS concentrations from 2.6 to 4.6 wt%, the response times of the hydrogels are 275, 300, and 337 s, respectively, which means that the response time increases with the crosslinking degree. After comprehensive consideration of mechanical strength and response time, we choose 3.6 wt% as the BIS concentration in our hydrogel.

### 3.4. The Working Principle of Double-Layer Hydrogel Robot

**Figure 2:**
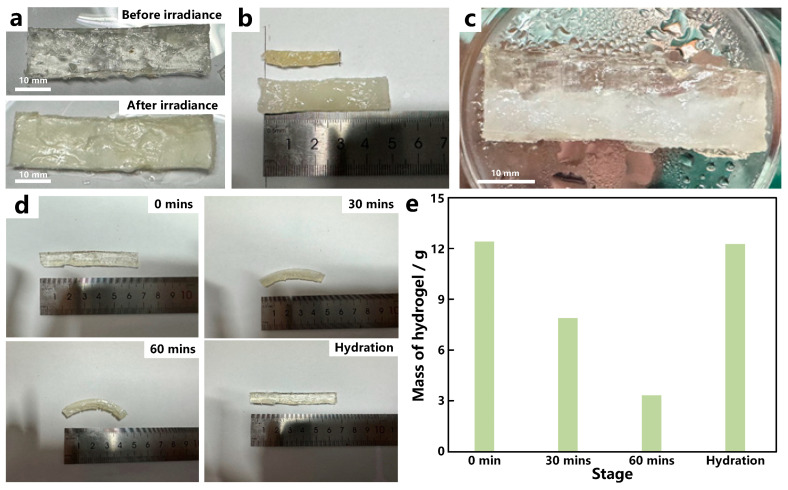
The working principle of double-layer hydrogel robot. Comparison of the top (**a**) and (**b**) bottom hydrogels before and after illumination; (**c**) physical picture of double-layered hydrogel; (**d**) bending process of double layer hydrogel under light; (**e**) masses of hydrogel at different stages.

The robot contains double hydrogel layers, which have significant differences in thermo-responsiveness. The working hydrogel robot relies on the anisotropy in thermo-responsiveness between the top and bottom layers. Figure 2a presents the physical picture of the top layer before and after light irradiance. Little dimension change was found for the top layer after light irradiance (1 W·m^−2^ for 1 h) except for the color changing from transparent to milky white due to the phase change of PNIPAm hydrogel. While for the bottom layer, the dimension changes significantly after being illuminated, the size has been roughly reduced to half of its original size (shown in Figure 2b). Owing to the significant differences between the two layers in response to light irradiance, it is expected that an obvious bending may occur under light irradiance when these two layers stick together. The combined layers using IDP technology are shown in Figure 2c. 

To verify this idea, we studied the deformation of the prepared double-layer hydrogel under light, and the working processes are illustrated in Figure 2d. The initial conformation of the hydrogel is straight, and the bending degree of hydrogel gradually increases with the increase in illumination time. The bending angle is 15 degrees at 30 min and 30 degrees at 60 min, which proves that the double-layer hydrogel prepared by us can effectively realize bending. When water is dropped on the surface of the hydrogel, the hydrogel can absorb the water quickly and the hydrogel returns to its initial conformation. Due to the bending and restoration with the change in temperature, the hydrogel can be used as the material of robots.

The driving force of bending action is the transition in swelling and collapse of the hydrogel when crossing LCST, so the hydration and dehydration processes of the hydrogel under irradiance are detected by measuring the mass of the hydrogel at different stages (Figure 2e). The initial mass of the hydrogel is 12.53 g. As the light radiates, the temperature increases gradually and the dehydration of the hydrogel occurs when the temperature is higher than LCST. After 30 min irradiance, the mass of the hydrogel reduces to 8.12 g and further reduces to 3.46 g after 60 min irradiance. When the light is off and the water is dropped on the surface of the hydrogel, hydration occurs, and the hydrogel mass returns to 12.52 g, which is almost the same as its initial mass.

**Figure 3:**
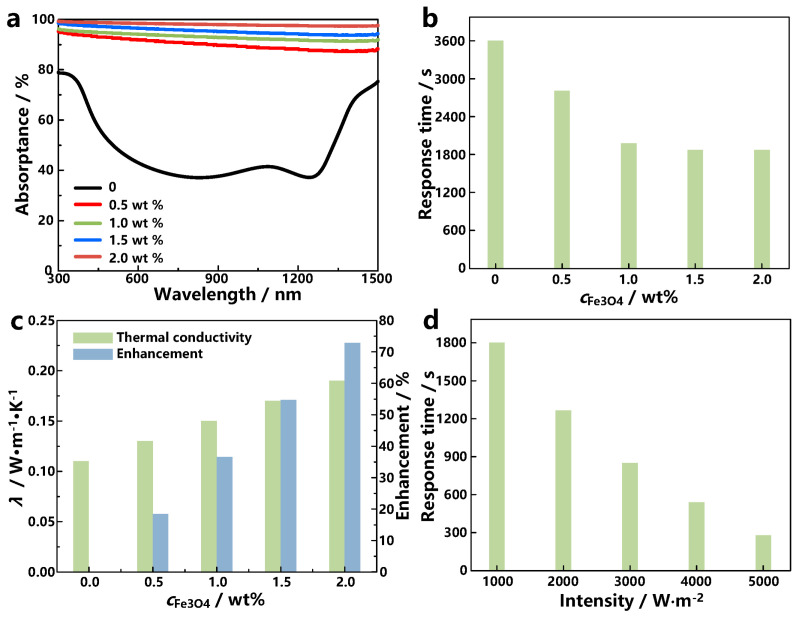
(**a**) Light absorptance of hydrogels at different Fe_3_O_4_ concentrations; (**b**) response time of hydrogel bending 30 degrees at different concentrations; (**c**) thermal conductivity and enhancement of hydrogels with different Fe_3_O_4_ loading; (**d**) response time of hydrogel with 2.0 wt% Fe_3_O_4_ loading bending 30 degrees under different light intensity.

Because the fundamental power of hydrogel deformation comes from the heat through photo-thermal conversion [23], it is also necessary to study the photo-thermal conversion characteristics of hydrogels. Here, Fe_3_O_4_ nanoparticles are used as light absorbers due to their ultra-high light absorption ability. Figure 3a presents the light absorptance of hydrogels with different Fe_3_O_4_ concentrations. Pure hydrogel has weak absorptance, particularly in the wavelength between 500 and 1400 nm. When adding Fe_3_O_4_ in the hydrogel, the absorptance across the entire spectral range has been enhanced, and the absorptance is as high as 98% when Fe_3_O_4_ concentration is 2 wt%. The better the light absorption performance, the shorter the response time of hydrogel deformation. Therefore, the response time of hydrogels with different Fe_3_O_4_ concentrations is also evaluated, and the time required for bending 30 degrees is shown in Figure 3b. For pour hydrogel, it should take about 1 h to bend 30 degrees, and the increase in Fe_3_O_4_ nanoparticles can significantly shorten response time. The response time of the hydrogel with 0.5 wt% Fe_3_O_4_ nanoparticles is just 2900 s. As the further increase in Fe_3_O_4_ nanoparticles, the response time continues to shorten and then converges around 1800 s when the temperature is 2.0 wt%. Therefore, this work adopts 2.0 wt% as Fe_3_O_4_ nanoparticles because little response time shortness can be achieved with higher Fe_3_O_4_ nanoparticles.

Besides the enhancement of light absorption, the addition of Fe_3_O_4_ nanoparticles can also improve the heat transfer ability of the hydrogel due to the high thermal conductivity of Fe_3_O_4_ nanoparticles. Figure 3c presents the thermal conductivity and its enhancement compared with pure hydrogel at different Fe_3_O_4_ concentrations. The thermal conductivity of dried pure hydrogel is as low as 0.11 W·m^−1^·K^−1^, which is not conducive to the transfer of heat concentrated on the surface of the hydrogel to the interior. The thermal conductivity can be significantly enhanced by adding Fe_3_O_4_ nanoparticles, and the thermal conductivity enhancement is approximately proportional to Fe_3_O_4_ concentration. The thermal conductivity and enhancement are as high as 0.19 W·m^−1^·K^−1^ and 72% when Fe_3_O_4_ concentration is 2.0 wt%. 

Meanwhile, there is no doubt that light intensity also has an impact on response time because the light intensity directly determines the energy of the photo-thermal conversion of the hydrogel, so we also study the influence of light intensity on the response time of the hydrogel, and the response time at 2.0 wt% Fe_3_O_4_ concentration are presented in Figure 3d. The increase in light intensity can significantly decrease the response time, and at 5000 W·m^−2^, light intensity is only 300 s, which is about one-sixth of that at 1000 W·m^−2^. Therefore, the increase in light intensity can significantly shorten the response time of the hydrogel robot.

In the work of Wei et al. [18], they systematically recorded the response time of different stages. Under 7000 W·m^−2^ NIR power, the bending and unbending times are 320 and 520 s, respectively. For our hydrogel robot, the bending and unbending times are 300 and 360 s under 5000 W·m^−2^ solar power, respectively, which indicates that our robot has a faster response compared with that of Wei et al. [18]. We attributed this priority to two factors. First, the light absorptance of our hydrogel robot is optimized by varying the concentration of light absorber (Fe_3_O_4_ nanoparticles), and the optimized hydrogel robot has excellent light absorption ability and ensures a fast bending response under irradiance. Second, the water absorption ability is significantly improved by adding SA or PVA hydrogels. With the addition of them, the hydrophilicity of PNIPAm hydrogel is highly enhanced (Figure 1c).

### 3.5. Performance of the Double-Layer Hydrogel Robot

**Figure 4:**
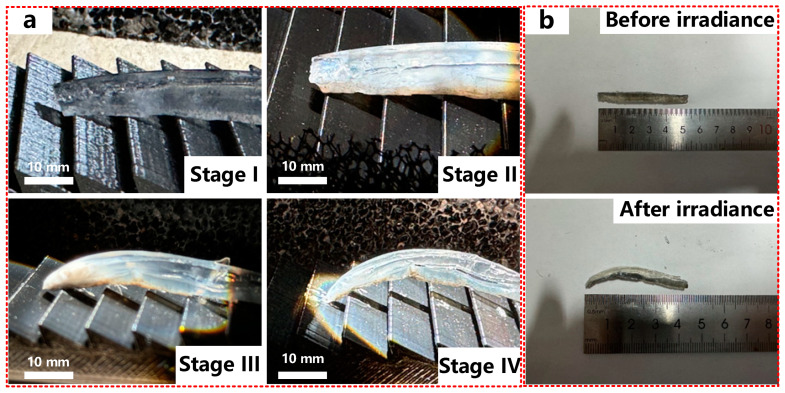
Performance of the double-layer hydrogel robot. (**a**) Moving process of hydrogel robot at different times; (**b**) conformation of hydrogel robot before and after light irradiance.

After verifying the effectiveness of the fabricated hydrogel, we constructed a hydrogel robot to evaluate its real moving performance on the platform. First, we measured the moving speed of the hydrogel with 2.0 wt% Fe_3_O_4_ concentration at 5 W·m^−2^ light intensity. We prepared a flat plate with ratchet structures as the moving plane of the hydrogel robot. The teeth of the ratchet structures are biased in one direction so that the bent part can be embedded in the front of the serrated part when the hydrogel bends and drags the whole hydrogel robot forward. The different stages of the hydrogel moving forward are shown in Figure 4a, and a video of the work process is shown in Supplementary Materials. The hydrogel is straight in the initial stage (Stage I) and begins to bend under light irradiance (Stage II). When the bending degree is large enough, the leading edge of the hydrogel will jam the ratchet structures (Stage III). With the further increase in bending, the hydrogel will move forward as a whole (Stage IV). When the light is off, the temperature of the hydrogel decreases, and the hydrogel returns to its original state after about 6 min. These are the steps for hydrogel to complete a whole moving action. The initial length of the hydrogel is 5.4 cm, and that of the bending hydrogel is about 4.4 cm, which means that the hydrogel can move about 1 cm in a complete moving action (shown in Figure 4b). It is easy to conclude that the prepared hydrogel robot has a moving speed of 54.5 mm·h^−1^.

### 3.6. Suitable Working Environment

The working process of the hydrogel robot is divided into bending and unbending stages. The bending stage is related to the light–thermal conversion ability of the hydrogel, so the response time at this stage can be reduced by both light-absorptance and light-intensity enhancement, which are easy to achieve in most environments. While the process of unbending stage highly relies on the ambient temperature. The bent hydrogel should cool its temperature and absorb water, so a lower ambient temperature is helpful in the unbending stage. Therefore, the proposed hydrogel robot is more suitable in cold regions.

## 4. Conclusions and Outlooks

In summary, a biomimetic double-layered hydrogel robot was fabricated using the IDP method, which shows excellent moving performance under light irradiance. Fe_3_O_4_ nanoparticles doped in hydrogel enhance the light absorption and thermal conductivity of the hydrogel, which significantly improves the response speed of the hydrogel to light irradiance. Meanwhile, the synthesized hydrogel has excellent mechanical strength and can tolerate significant flexibility tests, and the two layers of hydrogel are closely bonded, which ensures its durability as it works. The difference in the thermal response of the two hydrogel layers makes them produce anisotropic shrinkage under light, which makes it possible for the hydrogel to bend. As a result, the synthesized hydrogel robot can achieve a high moving speed of 54.5 mm·h^−1^. This work is an attempt of the thermo-responsive hydrogel robot to walk on the ground, and there are still many areas that need to be further improved, such as the improvement in moving speed and the integrated design of the phase change process. This work may inspire the development of the next generation of soft robots with adaptive shape change characteristics and reveal further corresponding applications.

## Figures and Tables

**Table 1:** Detailed information on the experimental samples.

Sample	Abbreviation	Role	Specification or Purity	Supplier
N-Isopropyl acrylamide	NIPAm	Monomer of hydrogel	Purity is 98%, containing MEHQ	Aladdin
Polyvinyl alcohol	PVA	Addition of hydrogel	Purity is 99%	Aladdin
Sodium alginate	SA	Addition of hydrogel	Analytical Reagent	Aladdin
Potassium persulfate	PP	Addition of hydrogel	Purity is 99%	Aladdin
Tetramethylethylenediamine	TEMED	Accelerator	Purity is 99%	Aladdin
N,N′-methylenebisacrylamide	BIS	Crosslinker	Purity is 99%	Aladdin
Ferroferric oxide	Fe_3_O_4_	Solar absorber	Diameter is 50 nm	XFNANO
Deionized water	DI	Dissolve the sample	/	Self-made

## Data Availability

The original contributions presented in the study are included in the article, further inquiries can be directed to the corresponding author.

## Supplementary Materials

The following supporting information can be downloaded at: https://www.mdpi.com/article/10.3390/ma17153679/s1.
